# Supplementary material for: MetaRibo-Seq measures translation in microbiomes
Source: Nat Commun. 2020 Jun 29;11:3268. doi: 10.1038/s41467-020-17081-z (PMC7324362; doi:10.1038/s41467-020-17081-z)
Supplement: Supplementary file 10 — Supplementary Data 7 [file 41467_2020_17081_MOESM10_ESM.zip › File2/Confidence_High_Taxonomy/307418_out.krona.html]

Javascript must be enabled to view this page.

members
magnitude
magnitudeUnassigned
count
unassigned
taxon
rank

307418\_out

2

superkingdom
2
2

1239
2
phylum

class
186801
1

order
1
186802

1
31979
family

genus
1
1485

1
1262840
species

SRS014404\_contig\_number\_7750

class
91061
1

order
1
1385

1950264
1
species

SRS075947\_contig\_number\_4600
